# Microbial Aetiology, Antibiotic Susceptibility and Pathogen-Specific Risk Factors for Udder Pathogens from Clinical Mastitis in Dairy Cows

**DOI:** 10.3390/ani11072113

**Published:** 2021-07-16

**Authors:** Anna Duse, Karin Persson-Waller, Karl Pedersen

**Affiliations:** Department of Animal Health and Antimicrobial Strategies, National Veterinary Institute, 751 89 Uppsala, Sweden; anna.duse@sva.se (A.D.); karin.persson-waller@sva.se (K.P.-W.)

**Keywords:** mastitis, antibiotic resistance, dairy cow, risk factors, Sweden

## Abstract

**Simple Summary:**

Mastitis is among the diseases in dairy cows that most often require antibiotic treatment. In order to maintain optimal treatment, it is important to have updated knowledge about the causative agents and their antibiotic resistance patterns. This investigation aimed to reveal the most important bacterial pathogens and their resistance patterns in Sweden, and we also identified some risk factors for infection with certain pathogens. The bacteria that were the most common causes of mastitis were, in descending order, *Staphylococcus aureus*, *Streptococcus dysgalactiae*, *Escherichia coli*, and *Streptococcus uberis*. Only a few Gram-positive bacteria were resistant to penicillin, and in general, the occurrence of antibiotic resistance was low. Therefore, the potential for antibiotic treatment of bovine mastitis in Sweden is good.

**Abstract:**

Mastitis is one of the most important infectious diseases and one of the diseases that causes the greatest use of antibiotics in dairy cows. Therefore, updated information on the bacteria that cause mastitis and their antibiotic susceptibility properties is important. Here, for the first time in over 10 years, we updated the bacterial findings in clinical mastitis in Swedish dairy cows together with their antibiotic resistance patterns and risk factors for each bacterial species. During the period 2013–2018, samples from clinical mastitis were collected, together with information on the cows and herds of origin. The samples were cultured, and a total of 664 recovered bacterial isolates were subjected to susceptibility testing. *Staphylococcus aureus* (*S. aureus*) was the most common pathogen and accounted for 27.8% of diagnoses, followed by *Streptococcus dysgalactiae* (*S. dysgalactiae*) (15.8%), *Escherichia coli* (*E. coli*) (15.1%), *Streptococcus uberis* (*S. uberis*) (11.4%), *Trueperella pyogenes* (*T. pyogenes*) (7.7%), non-*aureus* staphylococci (NAS) (2.8%), *Klebsiella* spp. (2.7%), *Enterococcus* spp. (1.3%), and *Streptococcus agalactiae* (*S. agalactiae*) (1.2%). Various other bacteria accounted for 2.6%. Staphylococci were, in general, susceptible to most antibiotics, but 2.6% of *S. aureus* and 30.4% of NAS were resistant to penicillin. No methicillin-resistant staphylococci were found. All *S. agalactiae* were susceptible to penicillin. Bimodal and trimodal MIC distributions for penicillin in *S. dysgalactiae* and *S. uberis*, respectively, indicate acquired reduced susceptibility in some isolates. The mostly unimodal MIC distributions of *T. pyogenes* indicate that acquired resistance does usually not occur in this species. Among *E. coli*, 14.7% were resistant to at least one antibiotic, most often ampicillin (8.7%), streptomycin (7.8%), or sulphamethoxazole (6.9%). *Klebsiella* spp. had low resistance to tetracycline (9.1%) but is considered intrinsically resistant to ampicillin. Pathogen-specific risk factors were investigated using multivariable models. *Staphylococcus aureus*, *S. dysgalactiae*, and *T. pyogenes* were more common, while *E. coli* was less common in quarters with more than one pathogen. *S. aureus* and *T. pyogenes* were mostly seen in early lactation, while *E. coli* was more common in peak to mid lactation and *S. dysgalactiae* in early to peak lactation. *Trueperella pyogenes* and *Klebsiella* spp. were associated with a previous case of clinical mastitis in the current lactation. *Staphylococcus aureus* was associated with tie stalls and *T. pyogenes* with loose housing. All pathogens except *E. coli* and *S. dysgalactiae* had a seasonal distribution. In conclusion, the aetiological agents for clinical bovine mastitis have remained relatively stable over the last 10–15 years, *S. aureus*, *S. dysgalactiae*, *E. coli* and *S. uberis* being the most important. Resistance to penicillin among Gram-positive agents was low, and in general, antibiotic resistance to other compounds was low among both Gram-positive and Gram-negative agents.

## 1. Introduction

Globally, mastitis is one of the most important infectious diseases in dairy herds [1]. The majority of clinical cases are caused by a limited number of specific pathogens, but in fact, a very large array of bacterial species may infect the udder. The panorama can also vary between countries and regions within each country. In order to implement suitable strategies against mastitis, it is important to understand the panorama of udder pathogens and to monitor trends over time. Mastitis is also by far the most important reason for antibiotic treatment in Swedish dairy herds, and about 60% of all antibiotics prescribed for parenteral treatment of dairy cows are for mastitis [2]. Hence, sustaining the efficacy of antibiotics is very important for dairy cow welfare and herd economics. Acquired antibiotic resistance in bacteria is, however, an increasing threat, and surveillance of antibiotic susceptibility of bacteria, including mastitis-causing pathogens, is recommended by the World Organisation for Animal Health (OIE) and several other organisations [3,4]. Results of such monitoring will guide therapeutic decisions and show possible trends, indicating a possible need for interventions regarding antibiotic use [4].

In Swedish dairy cattle herds, benzylpenicillin is by far the most prescribed antibiotic and drug-of-choice for most Gram-positive udder pathogens. Swedish veterinarians have, for several decades, complied with these recommendations, and it has been shown that benzylpenicillin, administered systemically in accordance with distributers’ recommendations, is used in over 80% of treatments for mastitis [2,5]. Although this antibiotic has a narrow spectrum, use of it may still enforce a selective pressure on certain bacterial species, which can increase the prevalence of antibiotic resistance [6]. Regular monitoring of the spectrum and susceptibility of udder pathogens is therefore warranted. The most recent nation-wide surveys were performed in 1994–1995 and 2002–2003 [7,8,9], and a timely update was therefore deemed necessary.

In the survey from 2002–2003, agent-specific risk factors were also sought [9]. Since the dairy herd structure has changed since then, with larger herds, more loose housing as opposed to tie stalls, and more cows milked in automatic milking systems, etc., it is possible that the importance of certain risk factors may have changed. Such information may be useful in future strategies combatting mastitis and is therefore of value to monitor.

The objectives of this study were to reveal the microbial panorama of udder pathogens associated with clinical mastitis and their antibiotic susceptibility in Swedish dairy cows and compare the results with previous investigations to identify important developments. Another objective was to investigate pathogen-specific risk factors.

## 2. Materials and Methods

### 2.1. Sampling

In August 2013, a project was initiated to continuously monitor the aetiology and antibiotic resistance in acute clinical mastitis in Swedish dairy cows. The project is a collaboration between the Swedish National Veterinary Institute (SVA) and Farm and Animal Health AB. Thirty-eight veterinary practices distributed throughout Sweden in the District Veterinary Organization collected milk samples. In Sweden, only veterinarians can prescribe antibiotics, and the veterinarian must visit the sick animal and make a diagnosis before prescribing such drugs. Each veterinary practice was encouraged to submit the first cases of clinical mastitis they diagnosed every month. This was to avoid selected cases being sampled for laboratory investigation. Only one quarter should be sampled. Samples should be taken from a lactating cow with observable abnormalities in milk in at least one quarter, with or without signs of swelling of the mammary gland or systemic illness. If more than one quarter was affected, only the most severely affected quarter should be sampled. When a case was identified, the responsible veterinarian collected individual milk samples aseptically from the selected quarter. At sampling, the following data about the cow and the herd were also registered: breed of the cow, udder quarter, lactation number, udder disease score based on cow somatic cell count (SCC) at the last three milk recordings [10], the result of California Mastitis Test (CMT; scored 1–5 where 1 = negative and 5 = strong gel formation), date of latest calving, if the cow had experienced another case of clinical mastitis during the current lactation, if she had been treated with antibiotics during the preceding 30 days or at drying off, the main housing system (tie-up or loose-housing) used in the herd, and if automatic milking systems (AMS) were used. Samples were collected from August 2013 to December 2018. The geographical location of the farms was defined from their regional Nomenclature des Unités Territoriales Statistiques (NUTS) location [11].

### 2.2. Culture of Samples

Storage and shipping of milk samples was carried out according to regular routines of the District Veterinary Organization. The samples, collected in sterile plastic tubes, were kept chilled from time of sampling to shipping and were sent at ambient temperature by over-night postal mail to SVA, Uppsala, Sweden on the day of collection. At SVA, bacteriological analysis was performed according to accredited routines (EN/ISO 17023). Briefly, 10µL were streaked onto blood agar (5% bovine blood) with esculin, incubated at 37 °C, and evaluated after 18–24 and 36–48 h. Growth on agar plates was initially characterized on the basis of colony morphology and α-, β-, or double haemolysis. Colonies of potential udder pathogens, except those considered as *S. aureus* based on colony morphology and double haemolysis, were put forward for genus and species identification with matrix-assisted laser desorption/ionization–time of flight mass spectrometry (MALDI-TOF MS) on a MALDI Biotyper (Bruker Daltonics, Bremen, Germany), using methods and spectra scores as previously described [12].

Evaluation of growth was performed according to accredited routines (EN/ISO 17023) at SVA. A milk sample was considered positive if at least one colony-forming unit (CFU) of *Staphylococcus* *aureus* (*S. aureus*)*,* non-*aureus* staphylococci (NAS), *Streptococcus* *agalactiae* (*S. agalactiae*), *Streptococcus dysgalactiae* (*S. dysgalactiae*), *Streptococcus uberis* (*S. uberis*), *Streptococcus parauberis* (*S. parauberis*), *Trueperella pyogenes* (*T. pyogenes*), *Pasteurella/Mannheimia* spp., *Escherichia coli* (*E. coli*), *Klebsiella* spp., or yeast was detected. For other bacteria, a positive classification required ≥ 5 CFU. If the growth of 2 colony types was detected, the milk sample was classified as having a mixed infection. If ≥ 3 colony types were detected, the milk sample was classified as contaminated. However, in line with National Mastitis Council (NMC) guidelines (2017), if low-level contamination, e.g., a few colonies of different morphologies, were found together with numerous colonies of one of the pathogens mentioned above, the sample was diagnosed as positive for the specific pathogen. Moreover, if growth of *S. aureus*, *S. dysgalactiae* and *T. pyogenes* was found in the same milk sample, the sample was diagnosed as positive for all three pathogens. This was in line with the evaluation used by Ericsson-Unnerstad et al. [9] and the laboratory routine (Table 1).

### 2.3. Antimicrobial Susceptibility Testing

Isolates of *S. aureus*, NAS, *S. uberis*, *S. dysgalactiae, S. agalactiae*, *T. pyogenes, E. coli* and *Klebsiella* spp. were subjected to susceptibility testing directly upon isolation by determination of minimum inhibitory concentration (MIC) using broth microdilution. Testing was performed essentially according to the recommendations of the Clinical and Laboratory Standards Institute [13] using VetMIC™ panels (National Veterinary Institute, Uppsala, Sweden) and cation-adjusted Mueller-Hinton broth (Becton Dickinson, Cockeysville, USA). The following modifications from CLSI were made: for the inoculum, the broth culture method was used for the testing of non-fastidious bacteria. Colony material was transferred into 5 mL of Mueller-Hinton broth and incubated at 35 °C for 3–5 h, after which 3–10 µL was transferred into 10 mL of Mueller-Hinton broth, which was subsequently used to inoculate the microtiter plates, with 50 µL in each well. For *T. pyogenes*, the direct colony suspension method was used. However, the inoculum was prepared using cation-adjusted Mueller-Hinton broth supplemented with 5% horse serum. The MIC panels were incubated at 35 °C, and *T. pyogenes* was read after 48 h, whereas other bacteria were read after 16–18 h. Antibiotics and test ranges are indicated in Table 2, Table 3, Table 4 and Table 5. For oxacillin susceptibility testing of staphylococci, the broth was supplemented with 2% NaCl. *Staphylococcus aureus* ATCC 29,213, *S. aureus* ATCC 25,923 and *E. coli* ATCC 25,922 were used as quality control reference strains. Staphylococci were examined for β-lactamase production using the “clover-leaf” method [14]. Isolates with MIC >2 mg/L for oxacillin or MIC >4 mg/L for cefoxitin were confirmed as methicillin-resistant staphylococci (MRS) through detection of the *mecA* or *mecC* genes, applying real-time PCR as previously described [15].

Isolates of *Enterobacterales* with cefotaxime or ceftazidime MIC above the respective EUCAST cut-off value, which depends on the species, were further analyzed with phenotypic confirmatory tests for production of extended spectrum beta-lactamases (ESBLs). The tests were performed with and without clavulanic acid in Sensititre EUVSEC2 microdilution panels (ThermoFisher, Oakwood Village, OH, USA) and interpreted according to the European Committee on Antimicrobial Susceptibility Testing (EUCAST) (http\\www.eucast.org, accessed on 14 July 2021). None of the isolates had a phenotype indicative of ESBL production, and further genotypic testing was not necessary.

Isolates were classified as susceptible (wildtype) or resistant (non-wildtype) based on EUCAST epidemiological cut-off values (ECOFFs). For *E. coli*, the ECOFF for trimethoprim-sulphadoxazole (>0.25 mg/L) could not be used since it was outside the range of tested concentrations. The same applied to the cut-off value for fusidic acid in *S. agalactiae* (> 32 mg/L). For *Klebsiella* spp., the cut-off value for cefotaxime (> 0.125 mg/L) would have falsely categorized one *K. pneumonia* isolate (cut-off > 0.25 mg/L) as non-wild-type, and hence the cut-off for *K. pneumonia* was used. The same applied to enrofloxacin, colistin, and tetracycline. The cut off-values used are provided in Table 2, Table 3, Table 4 and Table 5. Staphylococci were considered penicillin susceptible or resistant on the basis of the production of β-lactamase. When EUCAST ECOFFs were not available, isolates were not classified.

Isolates of *E. coli* with ciprofloxacin MIC > 0.06 mg/L and nalidixic acid MIC < 16 mg/L were selected for PCR detection of plasmid-mediated quinolone resistance genes, including *qnr*A, *qnr*B, *qnr*S, and *aac*(6′)-1b-cr, using the PCR assays described earlier [16].

### 2.4. Risk Factor Analyses

For the risk factor analyses, we excluded samples with no growth (*n* = 40), contaminated samples (*n* = 40), and samples without herd identity (*n* = 9). Quarter-level data were summarized at the cow level, yielding a 2-level dataset based on 755 udder quarter samples, 653 cows and 350 herds. A new variable, “number of diagnoses per quarter”, was generated, describing quarter-level features.

Descriptive statistics of all herd variables are given in Supplementary Table S1. To reduce the amount of missing data for the variables housing and AMS, loose housing was assumed in herds with AMS, and herds with tie stalls were assumed not to have AMS. The continuous variable, “days in milk” (DIM), was not linearly related to the log of the outcome (probability of isolating a certain pathogen) and was therefore categorized in terms of early (<50 DIM), peak (50–109 DIM), mid (110–209 DIM) and late lactation (>209 DIM). The odds of isolation of a certain pathogen from a sample, given the presence/absence of different risk factors, were estimated using logistic regression. The dependent variables in each model determined whether the cow was a case (1 = yes) or not (0 = no) with respect to *S. aureus*, NAS, *S. dysgalactiae*, *S. uberis*, *E. coli*, *Klebsiella* spp., or *T. pyogenes*, respectively, defining seven different mastitis outcomes. Each cow was represented once in each model, either as a case or a non-case. If more than one bacterial species was isolated, the cow was considered a case for each species and otherwise as a control. Non-cases in each model were cows with clinical mastitis caused by pathogens other than the one of interest.

There was a substantial number of missing values in the risk factor variables, ranging from 0 to 25% for different variables. Thus, each outcome was modelled twice (1) using only records with no missing data (complete case analysis, CC) and (2) using all records after multiple imputations of missing data (MI). Multiple imputation is a statistical technique for handling missing data. It has been shown multiple imputation analyses generally produce less biased results than complete case analyses [17]. Multiple imputation of missing values was carried out using the outcome variable and all risk factor variables, for which data were missing. Chained equations were used, and categorical predictors were imputed using a multinomial logit function, ordinal predictors using an ordered logit function and dichotomous predictors using a logit function. For each outcome, twenty imputed data sets were generated, and diagnostic plots comparing the distribution of the observed values with the imputed values were examined for the selected predictor variables.

Each herd was represented on average 1.86 times, hence assuming a limited clustering effect, and repeated observations within herd were accounted for using the cluster option (Stata). For each outcome and model (CC or MI), univariate associations (at *p* < 0.15) were used to identify candidate variables for the multivariable models. All two-way interactions between candidate predictors were included in the initial multivariable models and tested for significance. Non-significant (*p* > 0.05, likelihood ratio test) variables were eliminated in the multivariable analysis using a stepwise backward procedure. Model fit was assessed using the Hosmer and Lemeshow test, with data portioned in 10 deciles [18]. Stata, version 15 (StataCorp, College Station, TX, USA) was used for the statistical analyses.

## 3. Results

### 3.1. Bacteriological Findings

A total of 779 quarters from 738 cows were sampled. Thirteen samples had not been cultured, and the reason for not culturing was stated for two of them: delay in arrival to the laboratory. The remaining 766 udder quarter samples from 734 cows resulted in 835 microbial diagnoses (including no growth and contaminated samples). If a sample revealed growth of two pathogens, it was counted as two diagnoses. If the same bacterial species was diagnosed more than once from the same cow, only the isolate from the first sample was included. The other isolates (*n* = 12) were excluded due to risk of these being epidemiologically clustered. In total, 823 bacterial diagnoses from 755 udder quarters in 734 cows remained. One or more microbial species, based on culture and identification, were isolated from 675 (89%) of the quarter samples. From 611 (90.5%) of them, only one species was isolated; from 61 (8.9%) samples, two species were isolated; and from 4 (0.6%) samples, three species (*S. aureus*, *S. dysgalactiae* and *T. pyogenes*) were isolated. The distribution of the microbial diagnoses is shown in Table 1. For comparison, the results of two previous Swedish investigations are also presented [7,9]. Forty samples yielded no growth, while another 40 samples were contaminated. *Staphylococcus aureus*, *S. dysgalactiae*, *E. coli* and *S. uberis* were the most commonly detected species. *Trueperella pyogenes*, NAS, *Klebsiella* spp., *Enterococcus* spp., and *S. agalactiae* were found less often. Other bacteria were only found in a few cases each and together accounted for less than 1% each of the diagnoses.

### 3.2. Antibiotic Susceptibility

For the major mastitis-causing pathogens, antibiotic susceptibility results were available for 227 *S. aureus* isolates, 21 isolates of NAS, 120 *S. dysgalactiae*, 89 *S. uberis*, 9 *S. agalactiae*, 116 *E. coli*, 22 *Klebsiella* spp., and 60 *T. pyogenes*. Specific reasons why an isolate had not been tested or why susceptibility data were not in the laboratory database were not sought. For some antibiotics, susceptibility results were only available for a subset of the isolates due to changes in the composition of the susceptibility testing panels so that not all isolates were tested against all antibiotics depending on the antibiotics in the test panels. The results are provided as distributions of MICs, number of isolates tested and, when appropriate, cut-off values from EUCAST are available as percent resistant isolates in Table 2, Table 3, Table 4 and Table 5. In the tables, white fields indicate the test range for each antibiotic. MICs above the range are shown as the concentration closest to the range. MICs equal to or lower than the lowest concentration tested are shown as the lowest tested concentration. Bold vertical lines indicate the ECOFFs when available.

Sixteen (7.0%) of the 227 *S. aureus* isolates tested were resistant to one or more antibiotic. Penicillin resistance in *S. aureus*, i.e., β-lactamase production, occurred in six isolates (2.6%) (Table 2). All these isolates had MICs for penicillin > 0.12 mg/L. Five isolates (2.2%) were resistant to more than one antibiotic: one isolate to penicillin and oxacillin, one isolate to clindamycin and fusidic acid, one isolate to fusidic acid and tetracycline, one isolate to penicillin and fusidic acid, and one isolate to fusidic acid, kanamycin, and tetracycline. Eleven (52.3%) of the 21 NAS isolates tested were resistant to one or more antibiotic. Seven (30.4%) of the 21 tested isolates were resistant to penicillin through β-lactamase production, and all of these, where MIC results were available, had MICs for penicillin > 0.12 mg/L (Table 2). Two isolates (9.5%) were resistant to more than one antibiotic: one isolate to penicillin, clindamycin, fusidic acid, and erythromycin (not tested for oxacillin susceptibility) and one isolate to clindamycin, fusidic acid, erythromycin, and oxacillin. Bimodal distribution of MIC could be seen for cephalotin and trimethoprim. One isolate of *S. aureus* and one of NAS had MIC > 2 mg/L for oxacillin on testing at 37 °C. Neither the *S. aureus* nor the NAS isolate carried a *mecA* or a *mecC* gene, as confirmed by PCR.

Due to the lack of EUCAST cut-off values for streptococci in most antibiotics, the results are difficult to evaluate. Of the six *S. agalactiae* isolates, two were resistant to tetracycline (Table 3), for which one was also at the high end of the penicillin (MIC = 0.12 mg/L) and oxacillin (MIC = 1 mg/L) distributions. MICs to penicillin were below the epidemiological cut-off values in *S. agalactiae*. For *S. dysgalactiae*, there are no breakpoints except for clindamycin, to which none were resistant. However, for one isolate, the MIC for erythromycin (2 mg/L), and for another, the MIC for penicillin (0.25 mg/L), was above the breakpoint for *S. agalactiae*. Additionally, bimodal distributions of MIC were observed for fusidic acid and oxacillin. For *S. uberis,* there are no cut-off values, but distributions of MICs for some antibiotics were trimodal (erythromycin, penicillin, and tetracycline) or bimodal (cefotaxime, clindamycin, gentamicin, kanamycin, and oxacillin), indicating various degrees of acquired resistance in some isolates (Table 3). Seventeen isolates had deviating high MICs for penicillin above 0.06 mg/L, of which one isolate had MIC = 4 mg/L (Table 3).

Among *E. coli*, 17 isolates (14.7%) were resistant to one or more antibiotics. Ten isolates (8.6%) were resistant to at least two, and 8 (6.9%) to three or more antibiotics (Table 4). The most common traits were resistance to ampicillin (8.6%), streptomycin (7.8%), or sulphonamides (6.9%) (Supplementary Table S2). Six isolates (5.2%) were resistant to all these antibiotics. Five (4.3%) isolates were resistant to ceftazidime, but none of the isolates displayed phenotypic evidence of the production of ESBLs and hence were not put forward for genotypic testing. One isolate was resistant to ciprofloxacin while susceptible to nalidixic acid and was hence tested for the presence of plasmid-borne quinolone resistance genes but was confirmed as negative by PCR. For *Klebsiella*, most isolates were resistant to ampicillin (95.4%) and a few also to tetracycline (9.1%), colistin (4.6%), or ciprofloxacin (4.6%) (Table 4). One isolate had elevated MIC for colistin (4 mg/L), florfenicol (32 mg/L), chloramphenicol (32 mg/L), nalidixic acid (16 mg/L), streptomycin (64 mg/L), and trimethoprim (4 mg/L). Bimodal distributions of MIC values indicate acquired resistance in some isolates to chloramphenicol, florfenicol, nalidixic acid, streptomycin, sulphamethoxazole, and trimethoprim. None of the isolates were resistant to 3rd generation cephalosporins, indicating that none produced ESBLs.

EUCAST ECOFFs have not been defined for *T. pyogenes*, but the MIC distributions indicate that most isolates had not acquired resistance to most of the tested antibiotics (Table 5). Exceptions from this were trimethoprim alone and in combination with sulphamethoxazole as well as tetracycline, where bimodal distributions were seen. Two isolates had deviating high MICs for penicillin over 0.06 mg/L.

### 3.3. Risk Factors

Potential risk factor variables (Supplementary Table S1) contained between 0 and 25% missing values, after excluding the variable udder disease score due to a large proportion of missing records (67%). All missing values were successfully imputed, and comparison of diagnostic plots and tabulation of imputed and original values showed good imputation. We used pseudo-R-squared to compare the predictive abilities of the CC and MI models (Supplementary Tables S3 and S4). In general, the two models produced similar results and had the same subset of risk factors, but when comparing their predictive abilities, CC was superior to the MI model for all outcomes except *S. aureus*, for which it made no difference. Both results are presented in the tables but only the results from the CC analysis will be discussed. For both *S. uberis* and NAS, none of the significant risk factors from the MI model had missing values, and hence CC analysis was performed on all records.

Risk factor analysis was performed on data on the microbial panorama from cows with clinical mastitis, and the results must therefore be interpreted accordingly. Hence, a significant risk factor is associated with a higher likelihood of isolating a certain pathogen, given that a cow is infected. Results from the final multivariable models, including both CC and MI, can be found in Supplementary Table S3 for *S. aureus*, *T. pyogenes* and *E. coli* and in Supplementary Table S4 for *S. dysgalactiae*, *S. uberis*, *Klebsiella* spp., and NAS. The variables year, parity, breed, antibiotic treatment during the previous 30 days, dry cow antibiotic treatment at the latest dry off, and AMS were not significant in any of the final models.

Samples were accepted from all regions of Sweden (Supplementary Table S1). However, it became clear that some regions were over-represented and others under-represented considering the distribution of dairy cows in the country [19]. Calculations of regional effects were therefore not taken into consideration.

The risk of isolating *S. aureus* was higher if the cow was housed in tie stalls rather than loose housing and if the cow was in early compared with mid lactation. *S. aureus* was also more common in quarters with two or more udder pathogens isolated compared with only one and in mastitis cases occurring during the late housing season (January to April) compared with the early housing season (September to December). The probability of isolating NAS was higher during the late housing and pasture (May to August) seasons than during early housing season.

*Streptococcus dysgalactiae* was more common in quarters with two or more udder pathogens and from cases in early and peak rather than in mid lactation. The probability of isolating *S. uberis* was higher in cases during the early rather than the late housing season.

The risk of isolating *E. coli* was higher in peak and mid lactation compared with early lactation but was less common in quarters with two or more udder pathogens. *Klebsiella* spp. was 3.6 times more common in cows that had had a previous case of clinical mastitis in the current lactation, but it was also more rarely isolated in the early housing season than during the pasture season.

*Trueperella pyogenes* was more frequently found together with one or two other udder pathogens than in isolation and was also more common in cows in early lactation than in later lactation stages. It was also more common if the cow was in loose housing than in tie stalls and if the cow had a previous case of clinical mastitis in the current lactation.

## 4. Discussion

By comparing reports from different countries, it seems as though each country or study has its own unique panorama. For example, clinical cases in Finnish and Canadian dairy farms are often caused by NAS species [20,21], whereas in Sweden, very few clinical cases were due to this group of bacteria. Additionally, in many other countries, environmental pathogens such as *E. coli* or *S. uberis* dominate, while *S. aureus* is less common [22,23,24,25]. Whether the differences between countries are related to management practices, such as the use/non-use of blanket dry cow therapy, or other factors is currently not known. However, the five most common pathogens in clinical mastitis, *S. aureus*, *E. coli*, *S. dysgalactiae*, *S. uberis* and NAS, are seen in most studies, including ours [12].

Compared with the latest Swedish surveys [7,9], small but significant differences in the panorama were demonstrated with a relatively larger proportion of *S. aureus*, smaller proportions of NAS and samples with no growth in the present study. The dominance of the species *S. epidermidis*, *S. chromogenes*, *S. simulans* and *S. haemolyticus* among the NAS species confirms results from other studies [26,27,28,29]. Interestingly, the proportion of samples with no growth was also substantially lower than in many other reports [20,21,30,31]. There are several reasons for no growth in clinical mastitis samples; it has, for example, been suggested that some cases are due to *E. coli* infections that have been cleared by the cow’s immune system [32] and that the freezing of milk samples may have a negative effect on the survival and growth of certain bacteria such as *E. coli* but not *S. aureus* [33]. Since this pathogen is less commonly isolated in Sweden than elsewhere, fewer samples may also be culture negative. The likelihood of a negative culture also depends on the stage of the infection and inflammation. In our investigation, only clinical cases were included, which may have increased the likelihood of a positive culture. Another hypothesis is that some of the no growth samples in other studies may be due to *Mycoplasma bovis* infections, a pathogen that is not detected with routine culture methods. However, recent studies indicate that *M. bovis* is uncommon in Swedish dairy herds [34].

Udder pathogens were isolated from milk samples collected from all parts of Sweden. However, over half of the cases were from the northern parts of Sweden, although the southern parts are the most densely cow-populated areas [19]. This is a weakness of the present study. However, the distribution of mastitis-causing pathogens was similar to the previous nationwide survey in Sweden, in which a representative sample of cows from all parts of Sweden was included during the period 2002–2003 [9]. We therefore believe that our results are representative of the real panorama of pathogens causing clinical mastitis in Swedish dairy herds, although the samples were not geographically evenly distributed over the country. The veterinarians were instructed to submit a sample from the first cases of mastitis they were called to in the month, and this was done to avoid any selection bias. On the other hand, when more than one quarter displayed clinical mastitis, they were instructed to sample the quarter showing the most severe symptoms. This was to increase the chance of detecting a pathogen during laboratory examination, but since, in most cases, clinical mastitis in more than one quarter of the same cow at the same time is caused by the same pathogen, this will have had little influence on the results.

Benzylpenicillin is the drug of choice for most cases of mastitis in Sweden, and most Gram-positive udder pathogens are also susceptible to this drug. In the present study, 2.6% of *S. aureus* were resistant to penicillin. This is lower (results not shown) than in the latest nationwide surveys in 2002–2003 where penicillin resistance was seen in 7.1% of isolates [8]. Comparisons between studies should be made with caution, but the results indicate that penicillin-resistant *S. aureus* are on the decline in Sweden [7,9]. This may be an effect of the recommendation to cull all cows with penicillin-resistant *S. aureus.* A decline in penicillin resistance in *S. aureus* was also recently reported from France during the period 2006–2016, although the proportion of resistant isolates (33.9%) was considerably higher than in our study [35]. Considerably higher levels of resistance to penicillin in *S. aureus* have also been reported from several other countries [36,37].

Although occurrence of penicillin resistance in NAS in the 2002–2003 study (12.5%) [8] was numerically lower than in the present study (30.4%), the difference was not statistically different (results not shown), and the limited number of isolates tested precludes conclusions on trends. On the other hand, the proportion of resistant NAS resembles that detected by Persson et al. [38] from subclinical mastitis cases in Sweden. Methicillin resistance was not found in any of the staphylococci, indicating that methicillin-resistant *S. aureus* (MRSA) are rare in cases of clinical mastitis, and hitherto, only ten sporadic cases have been confirmed as MRSA in Swedish dairy cows [39]. Keeping the cattle population free from MRSA and LA-MRSA depends on hygiene measures and biosecurity. In a recent study in Denmark, it was demonstrated that LA-MRSA in cattle herds—both dairy and veal—were caused by spill-over from pig production [40]. However, the Swedish pig herds are free of LA-MRSA, which eliminates this hazard for the cattle population. The proportion of isolates with elevated MIC values for penicillin was higher for the NAS isolates than for *S. aureus*. This is in agreement with the findings from a recent German study [41] and in a study of isolates from nine different EU countries [42]. With only 21 NAS isolates, our data are insufficient to determine any differences in resistance or MIC distributions between different NAS species. It can only be encouraged that such issues are taken up by VETCAST or other fora, so that proper MIC distributions and ECOFFs for the most important NAS can be established, e.g., for *S. chromogenes*, *S. simulans*, or *S. haemolyticus*. Only very few ECOFFs or TECOFFs are available for these species.

All *S. agalactiae* and the majority of *S. dysgalactiae* and *S. uberis* had low MICs to penicillin, indicating clinical susceptibility, but one isolate of *S. dysgalactiae* and seventeen of *S. uberis* had MICs 0.12–0.25 mg/L, which indicates some degree of acquired resistance. Although there seems to be a trend towards decreased susceptibility of *S. uberis* to some antibiotics, penicillin and erythromycin since the last survey [8], the proportion of deviating high MICs is still much lower than in other European countries [43]. The distribution of MICs for *S. uberis* was trimodal, and it has been shown that mutations in penicillin-binding protein gene 1 in *S. uberis* can be evoked by repeated exposure to penicillin, resulting in a manifold increase in MIC [6]. The same mutations have been demonstrated in isolates from clinical bovine mastitis [6]. The existence of isolates with elevated MIC values for penicillin has also been reported by other researchers [28,41,42]. Still, most isolates have MIC values below the concentrations obtained in the inflamed udder using the pharmaceutically recommended dosage [44]. However, one isolate had MIC of 4 mg/L, which was much higher than in any of the isolates from a European study [43]. It is currently unknown whether such strains with elevated penicillin MIC values might cause therapy failure, and further research and monitoring of penicillin susceptibility of *S. uberis* is therefore critical.

The proportion of *E. coli* resistant to quinolones was lower (1.7%) than in the last survey (3.1%) [8]. Although we cannot exclude that this may in part be attributed to the uneven geographical origin of the samples, a possible explanation is a change in prescription. In Sweden, the use of critically important antibiotics, including fluoroquinolones, for animals was strictly regulated in 2013 [45]. A susceptibility test showing that no other antibiotics are effective is now needed to prescribe these drugs in non-life-threatening cases of mastitis. This is probably the reason why the prescription of fluoroquinolones has decreased and the use of trimethoprim-sulphonamide combinations has increased at around the same time [2]. The proportion of isolates resistant to more than one antibiotic was numerically higher in the present survey, but comparison is questionable due to the different sets of antibiotics tested. Neither among *Klebsiella* spp. nor *E. coli* was it possible to demonstrate ESBL production. Only a few isolates of ESBL-producing *E. coli* from cattle origin have been confirmed in Sweden, and none were from udder infections, a situation that differs from many other countries [46]. *Klebsiella* is considered intrinsically resistant to ampicillin [47], but apart from this, most isolates were susceptible to other compounds.

There are no approved epidemiological breakpoints for *T. pyogenes*, which makes conclusions about susceptibility more difficult to reach. Most isolates had MIC values below the concentration of benzylpenicillin obtained in the inflamed udder using the pharmaceutically recommended dosage, indicating clinical susceptibility. Bimodal distributions to some antibiotics were seen, but overall, MIC values for *T. pyogenes* were low, which is in accordance with surveys in other countries [48,49,50]. However, in vitro susceptibility is not the same as in vivo, and it may still not be possible to obtain clinical cure with this antibiotic [51].

Overall, the results of the susceptibility tests are similar to the previous survey [8], and there are no other indications of major shifts in susceptibility. This is in accordance with conclusions by Oliver and Murinda [36]. These authors compared the results from several studies of antibiotic resistance in mastitis pathogens from different countries and concluded that although antibiotic use in dairy cows can contribute to increased resistance, they found no indications of any widespread emergence of resistance in mastitis pathogens.

Housing seemed to be an important factor in determining the microbial panorama. We found that *S. aureus* was associated with tie stall housing, which is in line with previous observations [9,31]. These authors also found *E. coli* to be more common in loose housing [9,31] and *S. uberis* in tie stalls [31], which could not be confirmed by the present investigation. On the other hand, we found that *T. pyogenes* was more common in loose housing. Given these results combined, it is likely that the distribution of udder pathogens will change if we move towards more loose housing herds in Sweden, but hitherto, major shifts have not been observed.

Another factor that plays an important role is the season of sampling, which affected *S. aureus, S. uberis, Klebsiella* spp., and NAS. The finding that NAS and *S. aureus* mastitis was more common during the late housing season confirmed the results from Koivula et al. [20]. Studies in Norway [52] and Canada [53] also found that NAS was more likely during the late indoor season. In a study by Østerås et al. [52], *S. aureus* was most prevalent during the summer months, which is in contrast with the observations from our previous Swedish study [9], which related the higher isolation rate during the winter season to increased transmission indoors. However, with more loose housing, in which the transmission patterns are more similar throughout the year, it is more difficult to attribute seasonal differences to housing. Additionally, we could not see a sharp line between the indoor and pasture season regarding *S. aureus* isolation, and seasonal effects may instead be due to differences in climatic conditions over the year, which was also suggested by Koivula et al. [20]. Season also affected the probability of isolating *S. uberis*, and we saw a lower likelihood during the early housing season compared with the pasture and late housing season. This is in line with Lundberg et al. [54] and Makovec and Ruegg [55] but differs from other reports on seasonal differences for *S. uberis* [8,18,52,53]. *Klebsiella* spp. was more common during the pasture (summer) season, similar to previous findings [55]. Compared with the winter indoor season, during the summer, cows are more affected by adverse weather and sudden weather changes such as thunderstorms, which have been shown to predispose to coliform mastitis outbreaks [56]. Additionally, *Klebsiella* spp. seem to thrive better in bedding material during the summer compared with the winter [57]. We have no firm explanation as to why seasonal effects differ between different studies, and there may be other confounding factors that are masked by season, such as climate or whether cows are housed indoors or outdoors.

Consistent with the results of Persson Waller et al. [58], *S. aureus* and *T. pyogenes* peaked in early lactation, and we assume that these intramammary infections are predominantly persistent rather than new infections. It has been shown that many cows with *S. aureus* intramammary infection in early lactation were already infected at drying off, some despite treatment with dry cow antibiotics [59]. Cows are often immunocompromised around parturition [60], and it may be speculated that this may precipitate a persistent, subclinical infection into clinical mastitis. *Trueperella pyogenes* is often referred to as “dry cow mastitis”, and infection occurred more readily and with more severe clinical signs during the dry period than during lactation in an experimental study [61]. In the same study, the infection was rarely eliminated during the dry period and often persisted into the next lactation. In contrast, we found that *E. coli* was most commonly isolated in peak lactation, which is in line with Persson Waller et al. [58]. Likewise, the risk of *E. coli* infection seems to increase with milk yield [62]. The probability of *S. dysgalactiae* infection has been reported to decrease with increasing lactation stage [52], but in the present study, it occurred more commonly in early and peak than in mid lactation, which confirms previous results [58].

Both *Klebsiella* spp. and *T. pyogenes* were over three times more likely to be isolated in cows that had had a previous case of clinical mastitis. It can be assumed that a case of clinical mastitis makes the quarter more susceptible to a new intramammary infection [63]. Additionally, *T. pyogenes* may in some cases require a concomitant infection with another pathogen to cause clinical signs [61,64]. Likewise, we found that *T. pyogenes* was almost 18 times more likely to be isolated together with another pathogen. Additionally, *S. dysgalactiae* and *S. aureus* were more likely to be isolated together with another bacterium than others, and the results are in line with the previous survey [9]. These three bacteria were the only combination seen in quarters with three udder pathogens in the present study (results not shown). Co-infections of *S. dysgalactiae* and *T. pyogenes* are commonly seen in quarters from Swedish cows with mastitis [54,64], as are combinations of *S. aureus* and *S. dysgalactiae* [54]. *Escherichia coli*, on the other hand, was more often found in monoculture in this study, which is in contrast with the previous Swedish survey [9].

## 5. Conclusions

The most common pathogens found in cases of clinical mastitis were *S. aureus*, *E. coli*, *S. dysgalactiae* and *S. uberis*. In general, these pathogens were mostly susceptible to antibiotics commonly used in Sweden. Very few *S. aureus* were resistant to penicillin, while some isolates of *S. uberis* had an elevated MIC value due to penicillin, something that may offer therapeutic challenges. Housing, season, and history of previous cases of clinical mastitis were all identified as risk factors, depending on bacterial species.

## Figures and Tables

**Table 1:** Distribution of 823 microbial diagnoses from 755 udder quarters from cows with clinical mastitis (per cent, 95% confidence interval (CI) and number of diagnoses).

Diagnosis	2013–2018 (*n* = 823)			2002–2003 (*n* = 1056) Ericsson Unnerstad et al. 2009 [9]		1994–1995 (*n* = 837) Nilsson et al. 1997 [7]	
	*n*	%	CI	*n*	%	*n*	%
*Staphylococcus aureus*	229	27.8	24.7–31.0	225	21.3	181	21.6
Non-*aureus* staphylococci ^1^	23	2.8	1.8–4.2	65	6.2	35	4.2
*Streptococcus dysgalactiae*	130	15.8	13.4–18.5	165	15.6	113	13.5
*Streptococcus uberis*	94	11.4	9.3–13.8	117	11.1	128	15.3
*Streptococcus agalactiae*	10	1.2	0.6–2.2	6	0.6		
Other streptococci	4	0.5	0.1–1.2	9	0.9	11	1.2
Enterococci	11	1.3	0.7–2.4	8	0.8	7	0.8
*Trueperella pyogenes*	63	7.7	5.9–9.7	64	6.1	77	9.2
*Escherichia coli*	124	15.1	12.7–17.7	168	15.9	134	16.0
*Klebsiella* spp.	22	2.7	1.7–4.0	44	4.2	19	2.2
Other Enterobacterales ^2^	12	1.5	0.8–2.5	9 ^1^	0.9	8	0.8
Other bacteria ^3^	21	2.6	1.6–3.9	16 ^2^	1.5	9	0.9
Contaminated	40	4.9	3.5–6.6	48	4.5	49	5.9
No growth	40	4.9	3.5–6.6	112	10.6	66	7.9

^1^*S. chromogenes* (5), *S. epidermidis (5)*, *S. haemolyticus* (5), *S. simulans* (3), *S. cohnii* (2), *S. hyicus* (1), un-identified (2). ^2^ *Enterobacter* spp. (4), *Citrobacter freundii* (2), *Serratia* spp. (3), *Proteus mirabilis* (2), *Yersinia* (1). ^3^ *Bacillus* spp. (3), *Helcococcus* spp. (3), *Lactococcus* spp. (2), *Pasteurella multocida* (2) *Corynebacterium* spp. (1), yeast (6),* Mannheimia haemolytica* (2), *Acinetobacter* spp. (1), Gram-positive rod (1).

**Table 2:** Number of isolates (No), resistance (per cent, 95% CI in brackets), and MIC distribution (%) for *Staphylococcus aureus* (*n* = 227) and non-*aureus* staphylococci (NAS) (*n* = 21) from clinical mastitis in dairy cows. White fields indicate test range for each antibiotic.

Substance	Species	No	Resistance (%)	Distribution (%) of MICs (mg/L)												
				≤0.03	0.06	0.12	0.25	0.5	1	2	4	8	16	32	64	>64
Cefoxitin	*S. aureus*	97	0 (0.0–3.7)						1.0	49.5	49.5					
	NAS	10	0				10.0			80.0	10.0					
Cephalothin	*S. aureus*	227	0 (0.0–1.6)			51.5	44.5	3.5	0.4							
	NAS	21	-			47.6	38.1	4.8	9.5							
Chloramphenicol	*S. aureus*	227	0 (0.0–1.6)								19.8	77.1	3.1			
	NAS	21	0 (0.0–16.1)							4.8	71.4	19.1	4.8			
Ciprofloxacin	*S. aureus*	227	0 (0.0–1.6)			21.6	55.1	22.5	0.9							
	NAS	21	0 (0.0–16.1)			57.1	38.1	4.8								
Clindamycin	*S. aureus*	227	0.9 (0.1–3.1)				99.1	0.9								
	NAS	21	14.3 (5.0–34.6)				85.7	4.8	4.8	4.8						
Enrofloxacin	*S. aureus*	97	-			46.4	49.5	4.1								
	NAS	10	-			70.0	30.0									
Erythromycin	*S. aureus*	227	0 (0.0–1.6)				56.0	34.8	9.2							
	NAS	21	9.5 (2.7–28.9)				71.4	19.1				4.8		4.8		
Fusidic acid	*S. aureus*	227	4.4 (2.1–8.0)				73.1	22.5	2.6	0.9	0.4	0.4				
	NAS	21	14.3 (3.0–36.3)				57.1	23.8	4.8		4.8	9.6				
Gentamicin	*S. aureus*	227	0 (0.0–1.6)					89.4	9.3	1.3						
	NAS	21	0 (0.0–16.1)					100.0								
Kanamycin	*S. aureus*	130	0.8 (0.0–4.2)				0.8	9.2	16.9	54.6	10.8	6.9	0.8			
	NAS	11	-				63.6	27.3	9.1							
Linezolid	*S. aureus*	62	0.0 (0.0–5.8)						14.5	82.3	3.2					
	NAS	5	0.0 (0.0–52.2)						60.0	40.0						
Oxacillin	*S. aureus*	165	0.6 (0.0–3.3)				45.5	23.6	25.5	4.9	0.6 ^1^					
	NAS	16	6.2 (0.2–30.2)				50.0	25.0	18.8		6.2 ^1^					
Penicillin ^2^	*S. aureus*	227	2.6 (1.0–5.7)	60.4	32.2	4.0	0.4	0.4	0.4	0.9	0.9	0.4				
	NAS	23	30.4 (13.2–52.9)	33.3	28.6		9.5	9.5	9.5		9.5					
Tetracycline	*S. aureus*	227	0.9 (0.1–3.1)					96.5	2.6	0.9						
	NAS	21	0 (0.0–16.1)					95.2	4.8							
Trimethoprim	*S. aureus*	227	8.8 (5.5–13.3)					20.7	41.0	29.5	7.5	1.3				
	NAS	21	-					28.6	14.3	19.1	19.1	9.5	9.5			
Trimethoprim-Sulphadoxazole	*S. aureus*	97	0 (0.0–3.7)			97.9	2.1									
	NAS	10	-			60.0	20.0	10.0	10.0							

^1^ 1 *S. aureus* and 1 NAS tested negative for the *mecA* and *mecC* gene. ^2^ No cut-off value given, classification according to beta-lactamase production.

**Table 3:** Number of tested isolates (No), resistance (per cent, 95% CI in brackets), and MIC distribution (%) for *Streptococcus agalactiae*, *Streptococcus dysgalactiae* and *Streptococcus uberis* from clinical mastitis in dairy cows. White fields indicate test range for each antibiotic.

Substance	Species	No	Resistance (%)		Distribution (%) of MICs (µg/L)											
				≤0.03	≤0.06	0.12	0.25	0.5	1	2	4	8	16	32	64	>64
Cefoxitin	*S. agal.*	2	-							100.0						
	*S. dysg.*	41	-				46.3	43.9	7.3	2.4						
	*S. uberis*	38	-				2.6	47.4	26.3	7.9	10.5	5.3				
Cephalothin	*S. agal.*	9	-			100.0										
	*S. dysg.*	120	-			96.7	1.7	0.8	0.8							
	*S. uberis*	89	-			74.2	12.4	11.2	2.3							
Chloramphenicol	*S. agal.*	9	-							100.0						
	*S. dysg.*	120	-					1.7	25.0	65.0	7.5	0.8				
	*S. uberis*	89	-						4.5	32.6	61.8	1.1				
Ciprofloxacin	*S. agal.*	9	0 (0.0–33.6)					77.8	22.2							
	*S. dysg.*	120	-				17.5	72.5	9.2	0.8						
	*S. uberis*	89	-			1.1	20.2	52.8	25.8							
Clindamycin	*S. agal.*	9	0 (0.0–33.6)				100.0									
	*S. dysg.*	120	0 (0.0–3.0)				100.0									
	*S. uberis*	89	-				96.6	1.1	1.1						1.1	
Enrofloxacin	*S. agal.*	2	-					50.0	50.0							
	*S. dysg.*	41	-			2.4	9.8	85.4	2.4							
	*S. uberis*	38	-				15.8	68.4	15.8							
Erythromycin	*S. agal.*	9	0 (0–33.6)				100.0									
	*S. dysg.*	120	-				99.2			0.8						
	*S. uberis*	89	-				96.7	1.1				1.1		1.1		
>Fusidic acid	*S. agal.*	9	-								22.2	78.8				
	*S. dysg.*	120	-			0.9		3.3	47.5	0.8	5.8	1.7				
	*S. uberis*	89	-					1.1	1.1	23.6	51.7	22.4				
Gentamicin	*S. agal.*	9	-							44.4	44.4	11.1				
	*S. dysg.*	120	-					43.4	40.0	11.7	2.5	0.8	0.8	0.8		
	*S. uberis*	89	-					21.4	6.7	19.1	27.0	15.7	9.0	1.1		
Kanamycin	*S. agal.*	7	-										14.3	57.1	28.6	
	*S. dysg.*	79	-					6.3	2.5	22.8	20.4	25.3	7.6	5.1		
	*S. uberis*	51	-				5.9	9.8		7.8	5.9	27.5	29.4	11.8	1.9	
Linezolid	*S. agal.*	0	-													
	*S. dysg.*	24	-						100.0							
	*S. uberis*	24	-						50.0	50.0						
Oxacillin	*S. agal.*	9	-				66.7	22.2	11.1							
	*S. dysg.*	96	-				95.9	1.0	2.1		1.0					
	*S. uberis*	65	-				86.1	1.5	3.1	6.2	3.1					
Penicillin	*S. agal.*	9	0 (0.0–33.6)	44.4	44.4	11.1										
	*S. dysg.*	120	-	97.5	1.7		0.9									
	*S. uberis*	89	-	75.3	5.6	16.9	1.1				1.1					
Tetracycline	*S. agal.*	9	22.2 (2.8–60.0)					77.8				11.1			11.1	
	*S. dysg.*	120	-					8.4	10.0	49.2	27.5	2.5	0.8	0.8		0.8
	*S. uberis*	89	-					95.5	2.3			1.1			1.1	
Trimethoprim	*S. agal.*	9	-							33.3	55.6	11.1				
	*S. dysg.*	120	-					24.2	48.3	22.5	4.2		0.8			
	*S. uberis*	89	-					10.1	61.8	23.6	4.5					
Trimethoprim-Sulphadoxazole	*S. agal.*	2	-			50.0	50.0									
	*S. dysg.*	41	-			85.4	14.6									
	*S. uberis*	38	-			78.9	21.1									

**Table 4:** Number of tested isolates (No), resistance (per cent, 95% CI in brackets), and MIC distribution (%) for *Escherichia coli* and *Klebsiella* spp. isolated from cases of clinical mastitis in dairy cows. White fields indicate test range for each antibiotic.

Substance	Species	No	Resistance (%)	Distribution (%) of MICs (mg/L)																	
				≤0.016	0.03	0.06	0.12	0.25	0.5	1	2	4	8	16	32	64	128	256	512	1024	>1024
Ampicillin	*E. coli*	116	8.6 (4.2–15.3)							17.2	58.6	14.7	0.9		0.9		0.9	6.9			
	*Klebsiella*	22	95.4 (77.2–99.9)										4.6	18.1	45.4	22.7	4.6	4.6			
Ceftazidime	*E. coli*	116	4.3 (1.4–9.8)					80.2	15.5	3.5	0.9										
	*Klebsiella*	22	0 (0.0–15.4)					81.8	18.2												
Cefotaxime	*E. coli*	116	0 (0.0–3.1)			57.8	39.7	2.6													
	*Klebsiella*	22	0 (0.0–15.4)			81.8	13.6	4.6													
Chloramphenicol	*E. coli*	116	0 (0.0–3.1)								7.8	57.8	34.5								
	*Klebsiella*	22	-								27.2	63.6	4.6		4.6						
Ciprofloxacin	*E. coli*	116	1.7 (0.2–6.1)	10.3	69.8	18.1			0.9	0.9											
	*Klebsiella*	22	4.6 (0.1–22.8)	4.6	31.8	36.3	22.7		4.6												
Colistin	*E. coli*	116	6.0 (2.5–12.0)						25.0	42.2	26.7	6.0									
	*Klebsiella*	22	4.6 (0.1–22.8)						9.1	63.6	22.7	4.6									
Enrofloxacin	*E. coli*	28	0.0 (0.0–12.3)			100.0															
	*Klebsiella*	6	-			100.0															
Florfenicol	*E. coli*	116	0 (0.0–3.1)									41.4	53.5	5.2							
	*Klebsiella*	22	-									68.2	27.3		4.5						
Kanamycin	*E. coli*	88	2.3 (0.3–8.0)										97.7		2.3						
	*Klebsiella*	16	-										100.0								
Gentamicin	*E. coli*	116	0.9 (0.0–4.7)						79.3	18.1	1.7			0.9							
	*Klebsiella*	22	0 (0.0–15.4)						100.0												
Nalidixic acid	*E. coli*	116	0.9 (0.0–4.7)								47.4	47.4	4.3					0.9			
	*Klebsiella*	22	-								45.4	40.9	4.6	9.1							
Streptomycin	*E. coli*	116	7.8 (3.6–14.2)									50.9	37.9	3.5	0.9	2.6	4.3				
	*Klebsiella*	22	-									72.7	4.6	13.6	4.6	4.5					
Sulphamethoxazole	*E. coli*	116	6.9 (3.0–13.1)										19.0	50.9	23.3						6.9
	*Klebsiella*	22	-										9.1	22.7	45.4	18.2					4.6
Tetracycline	*E. coli*	116	4.3 (1.4–9.8)							64.7	31.0					2.6	0.9	0.9			
	*Klebsiella*	22	9.1 (1.1–29.2)							63.6	22.7	4.6		4.6	4.5						
Trimethoprim	*E. coli*	116	3.5 (0.9–8.6)				12.1	39.7	39.7	5.2					3.5						
	*Klebsiella*	22	-					31.8	50.0	13.6		4.6									
Trimethoprim-Sulphadoxazole	*E. coli*	28	-						92.9				7.1								
	*Klebsiella*	6	0.0 (0.0–45.9)						100.0												

**Table 5:** Number of tested isolates (No) and MIC distribution (%) for *Trueperella pyogenes* (*n* = 60) from clinical mastitis in dairy cows. White fields indicate test range for each antibiotic.

Substance	No	Distribution (%) of MICs (µg/L)												
		≤0.03	0.06	0.12	0.25	0.5	1	2	4	8	16	32	64	>64
Cephalothin	60			85.0	6.7	5.0	3.3							
Chloramphenicol	60					25.0	48.3	26.7						
Cefoxitin	18				100.0									
Ciprofloxacin	60			5.0	6.7	5.0	65.0	18.3						
Clindamycin	60				95.0	1.7	3.3							
Enrofloxacin	18					5.6	88.8	5.6						
Erythromycin	60				96.6	1.7	1.7							
Fusidic acid	60		21.7	23.3	35.0	18.3	1.7							
Gentamicin	60					43.3	20.0	33.3	1.7	1.7				
Kanamycin	42					4.8	28.6	42.8	21.4	2.4				
Linezolid	11						100.0							
Oxacillin	49			14.2	20.5	16.3	18.4	14.3	12.2	4.1				
Penicillin	60	86.6	10.0	1.7	1.7									
Tetracycline	60					86.6	5.0	1.7	1.7		5.0			
Trimethoprim	60					41.7	28.3	8.3	5.0	16.7				
Trimethoprim-Sulphadoxazole	17			11.8	11.8	29.4	5.9	5.9	35.4					

## Data Availability

The datasets generated and analysed during this study are available upon reasonable request. Bacterial isolates are available and can be requested provided an MTA is agreed.

## Supplementary Materials

The following are available online at https://www.mdpi.com/article/10.3390/ani11072113/s1, Table S1: Descriptive statistics of variables reflecting farm and cow characteristics, Table S2: Resistance phenotypes of *Escherichia coli* (*n* = 17) resistant to at least one antibiotic, Table S3: Final multivariate models with odds ratio (OR) estimates with 95% confidence intervals (CI) for the risk of isolating *Staphylococcus aureus, Trueperella pyogenes*, and *Escherichia coli* from cases of clinical mastitis in 653 Swedish dairy cows, Table S4: Final multivariate models with odds ratio (OR) estimates with 95% confidence intervals (CI) for the risk of isolating *Streptococcus dysgalactiae, Streptococcus uberis, Klebsiella* spp. and non-*aureus* staphylococci (NAS) from cases of clinical mastitis in 653 Swedish dairy cows.
