# Supplementary material for: Microbial Aetiology, Antibiotic Susceptibility and Pathogen-Specific Risk Factors for Udder Pathogens from Clinical Mastitis in Dairy Cows
Source: Animals (Basel). 2021 Jul 16;11(7):2113. doi: 10.3390/ani11072113 (PMC8300163; doi:10.3390/ani11072113)
Supplement: Supplementary file 1 [file animals-11-02113-s001.zip › animals-1303152-supplementary.pdf]

Table S1. Descriptive statistics of variables reflecting farm and cow characteristics for 653 cows with clinical mastitis in 350 Swedish dairy herds, from August 2013 to December 2018.

Variable	Category	Frequency (%)
Year	2013	68 (10)
	2014	127 (20)
	2015	70 (11)
	2016	106 (16)
	2017	111 (17)
	2018	171 (26)
Season	Early housing season (Sep-Dec)	274 (42)
	Late housing season (Jan-Apr)	237 (36)
	Pasture (May-Aug)	142 (22)
Region	East	111 (17)
	South including the islands	165 (25)
	North	377 (58)
Housing	Loose	396 (61)
	Tie stalls	226 (35)
	Unknown	31 (5)
AMS[1]	Yes	204 (31)
	No	329 (50)
	Unknown	120 (19)
Breed[2]	SH	325 (50)
	SR	247 (38)
	Other	29 (4)
	Unknown	52 (8)
Parity	1	118 (18)
	2	124 (19)
	3	120 (18)
	>3	128 (20)
	Unknown	163 (25)
Lactation stage	Early lactation (<50 days in milk)	273 (42)
	Peak lactation (50-109 days in milk)	76 (12)
	Mid lactation (110-209 days in milk)	97 (15)
	Late lactation (>209 days in milk)	72 (11)
	Unknown	134 (20)
Udder disease score[3]	0-2	122 (19)
	3-5	44 (7)
	6-9	52 (8)
	Unknown	435 (66)
Previous clinical mastitis	Yes	79 (12)
	No	530 (81)
	Unknown	44 (7)
Previous antibiotic treatment	Yes	19 (3)
	No	600 (92)
	Unknown	34 (5)
Dry cow antibiotic treatment	Yes	80 (12)
	No	361 (55)
	Not applicable	110 (17)
	Unknown	102 (16)
Number of species in the same quarter[3]	1	589 (90)
	2-3	64 (10)

[1]AMS = Automatic milking system.
[2]SH = Swedish Holstein, SR = Swedish Red.
[2]According to (Brolund, 1990).

Table S2. Resistance phenotypes of *Escherichia coli* (n=17) resistant to at least one antibiotic. "R" indicates resistance. Isolates were from cases of clinical mastitis in Swedish dairy cows.

Number of isolates	Resistance phenotype[1]								
	Sm	Su	Am	Tc	Na	Ci	Tm	Cz	Gm
1	R	R	R	R			R	R	
3	R	R	R				R		
2	R	R	R						
1	R	R							
1	R								
1	R		R	R					
1	R							R	
1		R	R	R	R	R			R
1			R						
2				R					
1						R			
2								R	

[1] Sm: streptomycin; Su: sulphamethoxazole; Am: Ampicillin; Tc: tetracycline; Na: nalidixic acid; Ci: ciprofloxacin; Tm: trimethoprim; Cz: ceftazidime; Gm: gentamicin.

Table S3. Final multivariate models with odds ratio (OR) estimates with 95% confidence intervals (CI) for the risk of isolating *Staphylococcus aureus, Trueperella pyogenes,* and *Escherichia coli* from cases of clinical mastitis in 653 Swedish dairy cows, during 2013–2018. The results are from the complete case analysis and the analysis using multiple imputed data of variables with missing values.

Variable	Levels	*S. aureus*[1]						*T. pyogenes*						*E. coli*					
		Complete case (R2=6.5%)			Imputed data (6.5%)			Complete case (R2=24.7%)			Imputed data (R2=22.3%)			Complete case (R2=6.1%)			Imputed data (R2=4.4%)		
	No. of obs (no. of cases)	512 (179)			653 (228)			510 (46)			653 (62)			518 (101)			653 (120)		
		OR	CI	p	OR	CI	p	OR	CI	p	OR	CI	p	OR	CI	p	OR	CI	p
No. of diagnoses per quarter	1	1.00	-	-	1.00	-	-	1.00	-	-	1.00	-	-	1.00	-	-	1.00		
	2-3	3.53	1.78; 7.00	<0.001	3.81	2.14; 6.80	<0.001	17.52	7.89; 38.93	<0.001	13.50	6.71; 27.16	<0.001	0.32	0.10; 0.95	0.041	0.33	0.13; 0.87	0.006
Geographic region	South including the islands	[1]												1.00	-	-	1.00		
	East													0.51	0.24; 1.09	0.083	0.43	0.23; 0.80	0.006
	North													0.44	0.26; 0.74	0.003	0.54	0.33; 0.87	0.014
Lactation stage	Early (<50 DIM[2])	1.00	-	-	1.00	-	-	1.00	-	-	1.00	-	-	1.00	-	-	1.00	-	-
	Peak (50-109 DIM)	0.58	0.33; 1.01	0.054	0.63	0.36; 1.12	0.116	0.12	0.02; 0.59	0.009	0.18	0.04; 0.81	0.026	2.59	1.41; 4.77	0.006	2.10	1.15; 3.84	0.016
	Mid (110-209 DIM)	0.39	0.22; 0.70	0.002	0.47	0.27; 0.81	0.007	0.34	0.12; 0.93	0.036	0.46	0.19; 1.14	0.094	2.41	1.28; 4.51	0.002	1.95	1.05; 3.65	0.035
	Late (>209 DIM)	0.65	0.37; 1.16	0.147	0.69	0.39; 1.20	0.187	0.18	0.05; 0.63	0.008	0.20	0.06; 0.67	0.011	1.62	0.81; 3.23	0.170	1.49	0.76; 2.92	0.241
Previous clinical mastitis	No							1.00	-	-	1.00	-	-						
	Yes							3.73	1.58; 8.81	0.003	3.74	1.64; 8.48	0.002						
Housing	Loose	1.00	-	-	1.00	-	-	1.00	-	-									
	Tie stall	2.11	1.40; 3.17	<0.001	1.87	1.29; 2.72	0.001	0.42	0.18; 0.96	0.040									
Season	Late housing season (Jan-Apr)	1.00	-	-							0.57	0.29; 1.11	0.098						
	Pasture (May-Aug)	0.66	0.41; 1.04	0.073							0.33	0.14; 0.78	0.011						
	Early housing season (Sep-Dec)	0.59	0.40; 0.87	0.007							1.00	-	-						

[1]According to [10]
[2]Empty fields represent variables that were not significantly associated with isolation of a certain pathogen, but included in the table due to its association to another pathogen
[3]DIM = days in milk.

Table S4. Final multivariate models with odds ratio (OR) estimates with 95% confidence intervals (CI) for the risk of isolating *Streptococcus dysgalactiae, Streptococcus uberis, Klebsiella* spp. and non-*aureus* staphylococci (NAS) from cases of clinical mastitis in 653 Swedish dairy cows, during 2013–2018. The results are from the complete case analysis and the analysis using multiple imputed data of variables with missing values.

Variable	Levels	*S. dysgalactiae*						*S. uberis*			*Klebsiella* spp.						NAS		
		Complete case (R2=12.9%)			Imputed data (R2=12.3%)			Complete case (R2=0.9%)			Complete case (R2=6.7%)			Imputed data (R2=6.6%)			Complete case (R2=3.3%)		
	No. of obs (No. of cases)	519 (105)			653 (128)			653 (92)			512 (12)			653 (22)			653 (23)		
		OR	CI	p	OR	CI	p	OR	CI	p	OR	CI	p	OR	CI	p	OR	CI	p
No. of diagnoses per quarter	1	1.00	-	-	1.00	-	-	[1]											
	2-3	12.29	6.41; 23.57	<0.001	11.43	6.50; 20.12	<0.001												
Geograhic region[2]	South including the islands							1.00	-	-									
	East							2.20	1.10; 4.39	0.025									
	North							1.46	0.79; 2.70	0.229									
Lactation stage	Early (<50 DIM[3])	2.39	1.16; 4.89	0.018	2.01	0.99; 4.08	0.052												
	Peak (50-109 DIM)	3.25	1.35; 7.81	0.009	2.62	1.16, 5.91	0.021												
	Mid (110-209 DIM)	1.00	-	-	1.00	-	-												
	Late (>209 DIM)	1.63	0.60; 4.44	0.340	1.42	0.54; 3.72	0.478												
Previous clinical mastitis	No										1.00	-	-	1.00	-	-			
	Yes										3.59	1.50; 8.61	0.004	3.27	1.34; 7.98	0.009			
Season	Late housing season (Jan-Apr)							1.00	-	-	0.42	0.16; 1.12	0.084	0.41	0.17; 0.99	0.048	3.60	1.09; 11.91	0.036
	Pasture (May-Aug)							0.94	0.51; 1.75	0.849	1.00	-	-	1.00	-	-	3.50	1.03; 11.89	0.045
	Early housing season (Sep-Dec)							0.56	0.32; 0.98	0.043	0.27	0.09; 0.85	0.025	0.24	0.08; 0.74	0.013	1.00	-	-

[1]Empty fields represent variables that were not significantly associated with isolation of a certain pathogen, but included in the table due to its association to another pathogen
[2]According to (European Union, 2011)

[3]DIM = days in milk
